# Supplementary figures and images for: Linking 16S rRNA Gene Classification to amoA Gene Taxonomy Reveals Environmental Distribution of Ammonia-Oxidizing Archaeal Clades in Peatland Soils
Source: mSystems. 2021 Aug 31;6(4):10.1128/msystems.00546-21. doi: 10.1128/msystems.00546-21 (PMC12338138; doi:10.1128/msystems.00546-21)

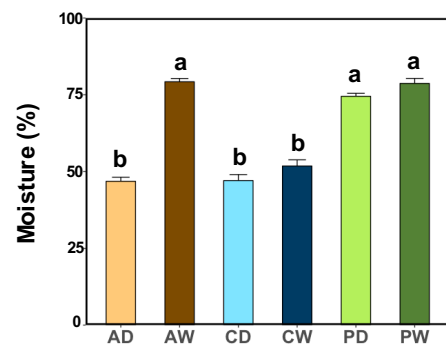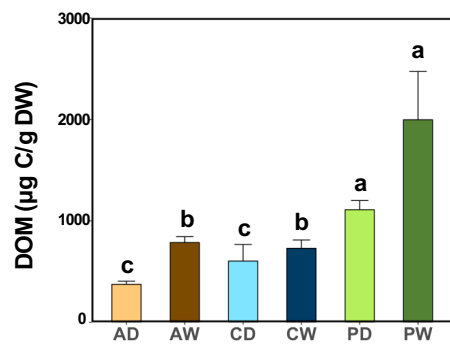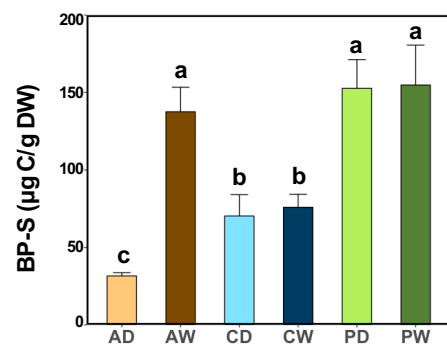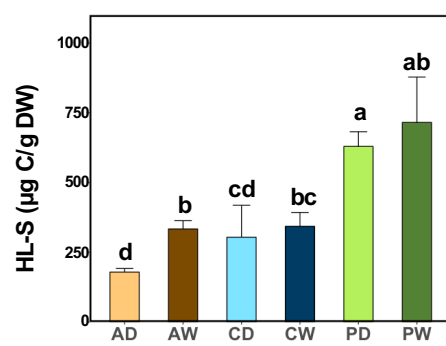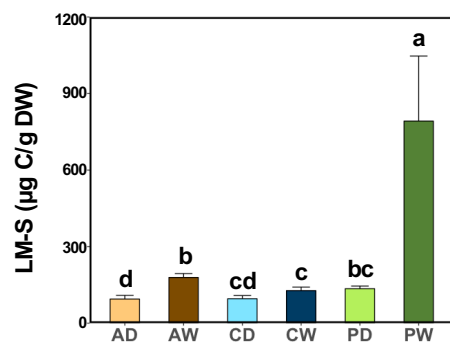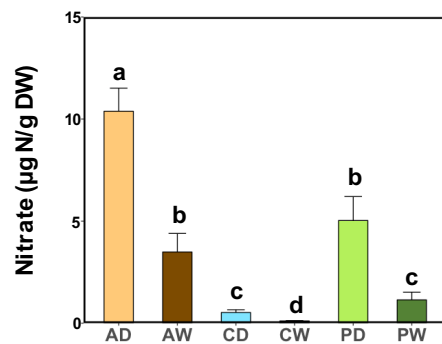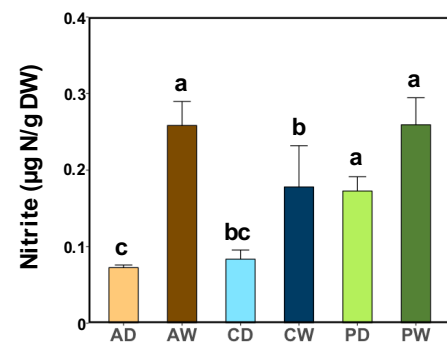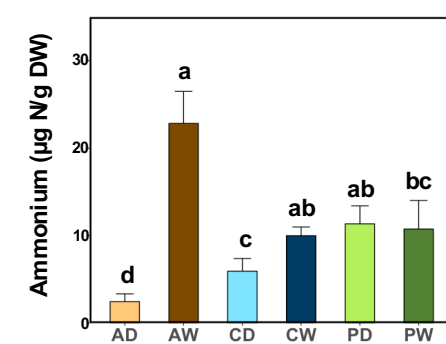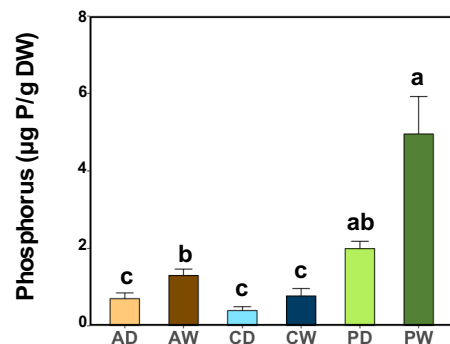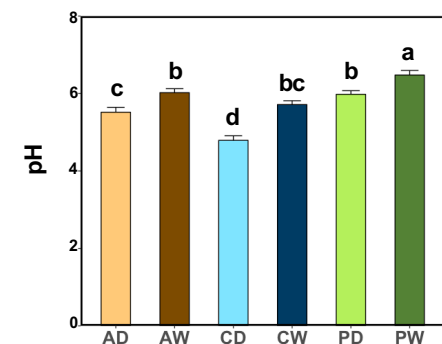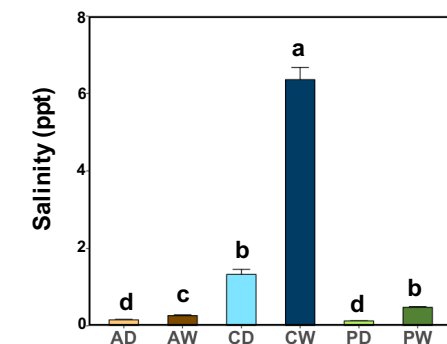

Supplement: FIG S1 [file msystems.00546-21-sf001.pdf]

A)

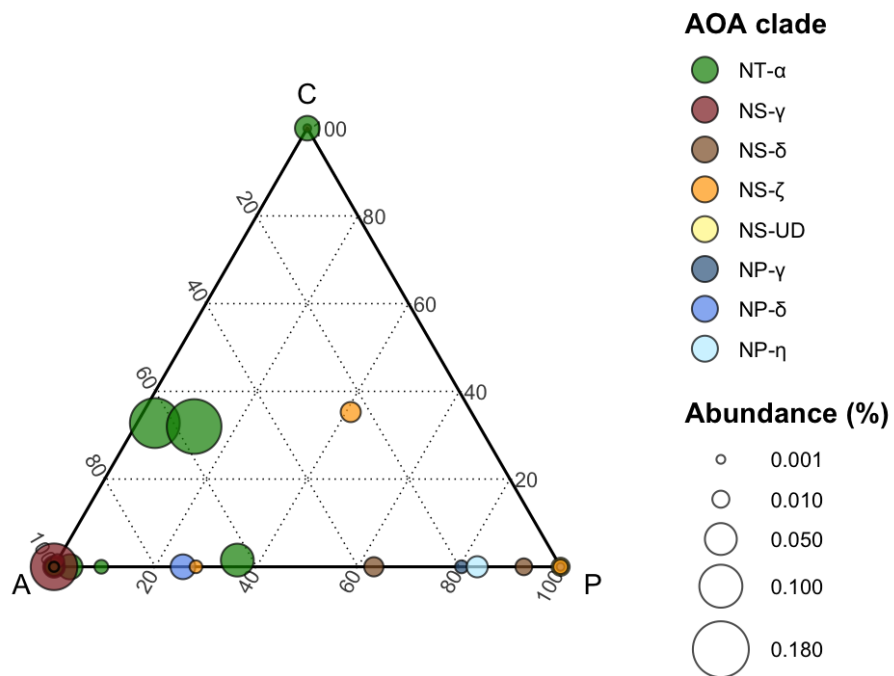

B)

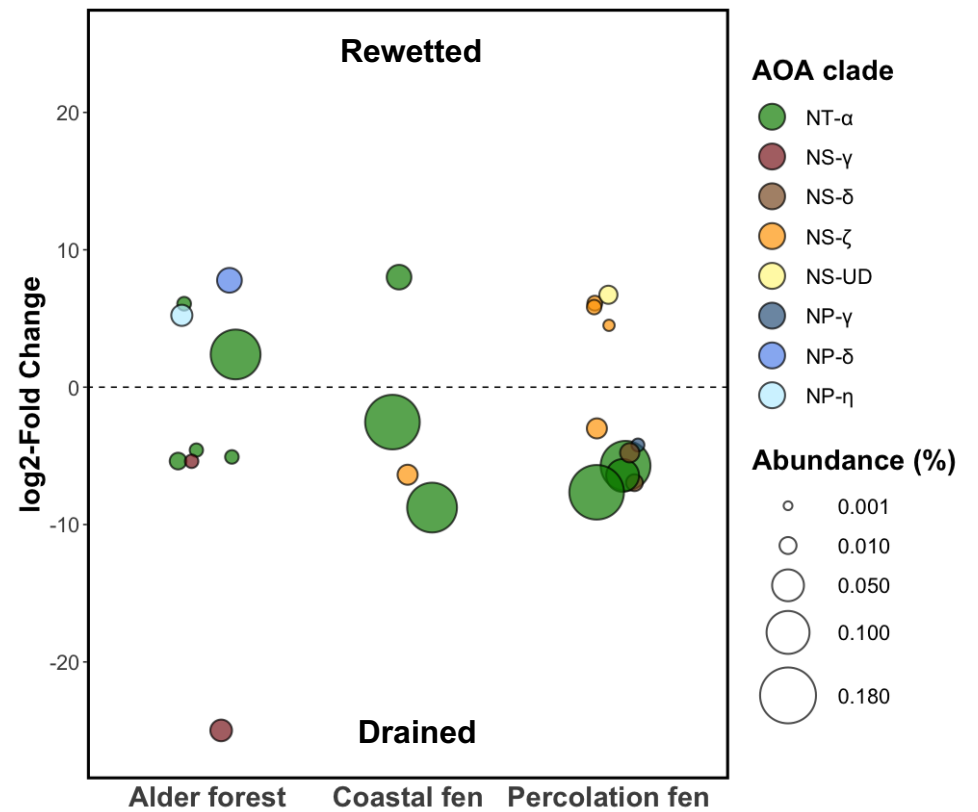

Supplement: FIG S2 [file msystems.00546-21-sf002.pdf]

A)

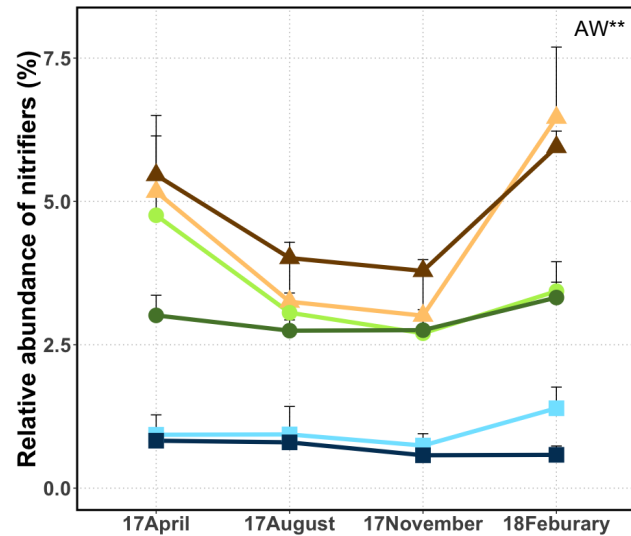

B)

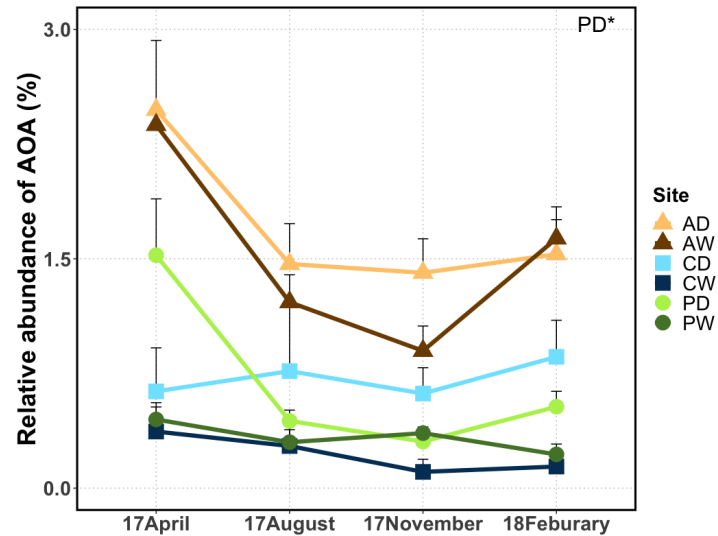

C)

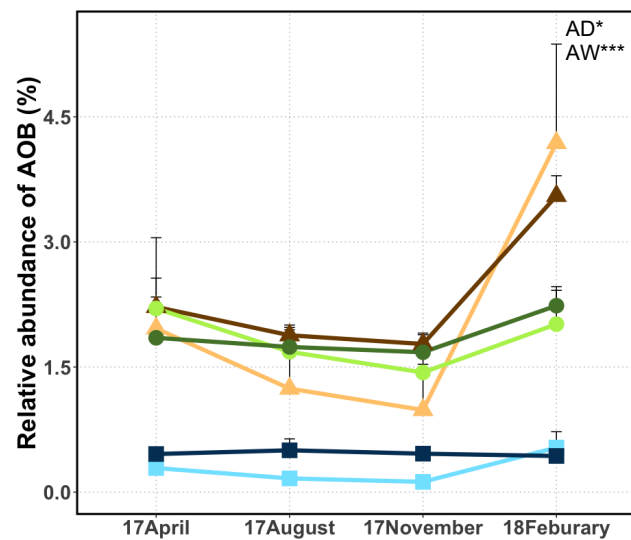

D)

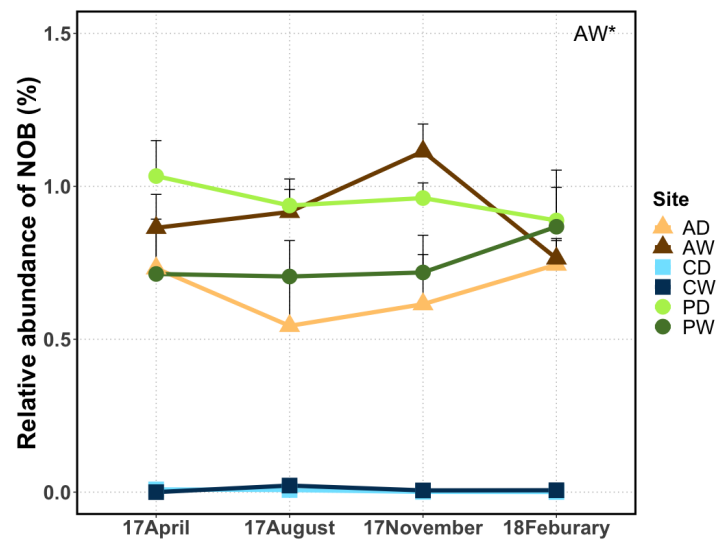

Supplement: FIG S3 [file msystems.00546-21-sf003.pdf]

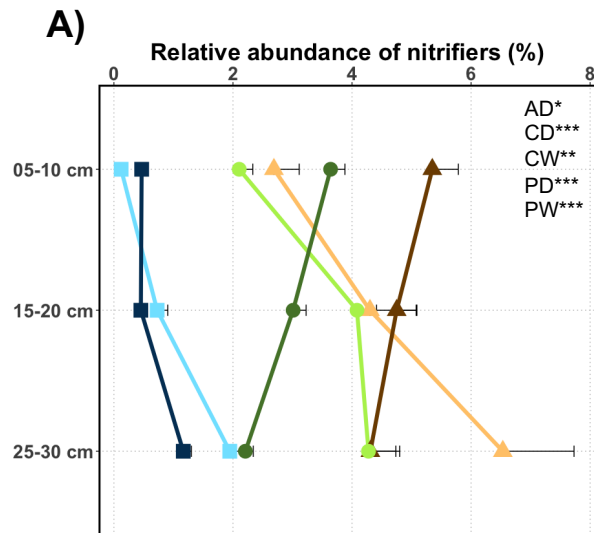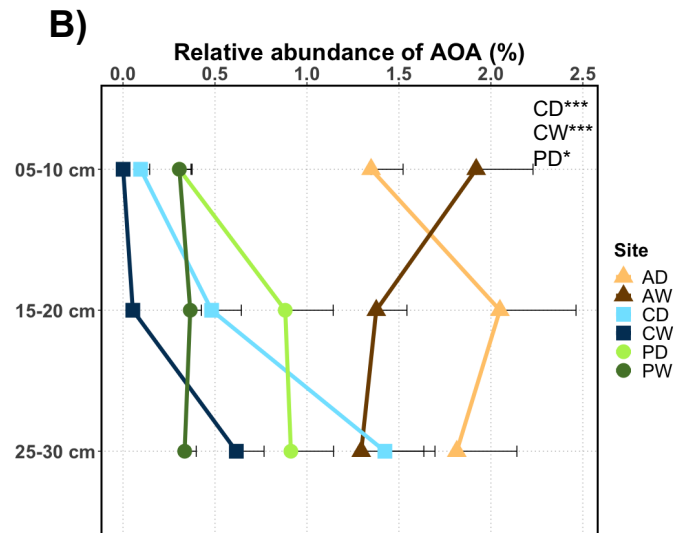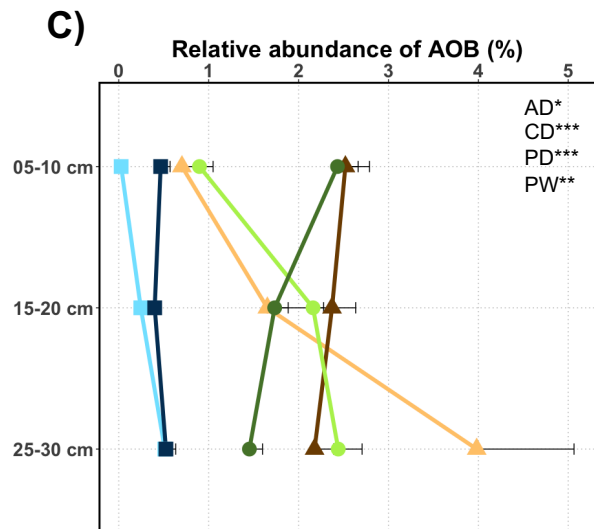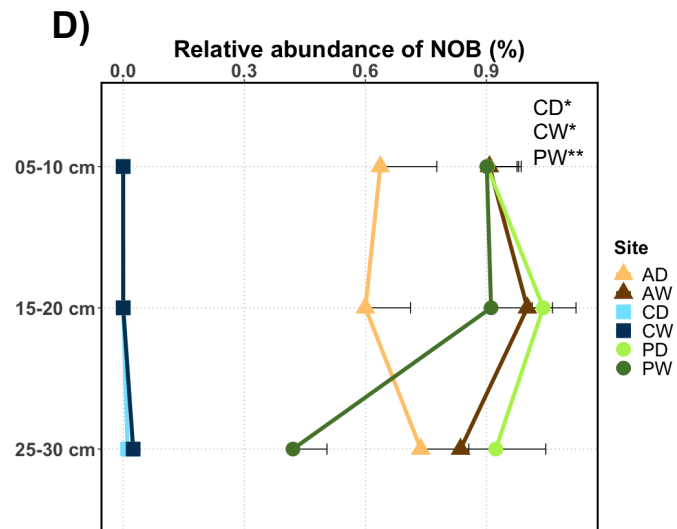

Supplement: FIG S4 [file msystems.00546-21-sf004.pdf]
